# Novel proteomics biomarkers of recurrent pregnancy loss reflect the dysregulation of immune interactions at the maternal-fetal interface

**DOI:** 10.3389/fimmu.2025.1621168

**Published:** 2025-08-28

**Authors:** Eszter Tóth, Máté Posta, Dániel Györffy, Orsolya Oravecz, Emese Farkas, Andrea Balogh, Claudia Escher, Magdalena Bober, András Szilágyi, Petronella Hupuczi, Lajos Veress, Olga Török, Sándor Nagy, Oliver Rinner, Offer Erez, Zoltán Papp, Nándor Ács, Nándor Gábor Than

**Affiliations:** ^1^ Systems Biology of Reproduction Research Group, Institute of Molecular Life Sciences, HUN-REN Research Centre for Natural Sciences, Budapest, Hungary; ^2^ Doctoral College, Károly Rácz Conservative Medicine Division, Semmelweis University, Budapest, Hungary; ^3^ Department of Bioinformatics, Semmelweis University, Budapest, Hungary; ^4^ Faculty of Information Technology and Bionics, Pázmány Péter Catholic University, Budapest, Hungary; ^5^ Doctoral School of Biology, Institute of Biology, ELTE Eötvös Loránd University, Budapest, Hungary; ^6^ Biognosys AG, Schlieren, Switzerland; ^7^ Maternity Private Clinic of Obstetrics and Gynecology, Budapest, Hungary; ^8^ Department of Pharmacology and Pharmacother, University of Debrecen, Debrecen, Hungary; ^9^ Department of Obstetrics and Gynecology, Faculty of Medicine, University of Debrecen, Debrecen, Hungary; ^10^ Department of Obstetrics of Gynecology, Széchenyi István University, Győr, Hungary; ^11^ Department of Obstetrics and Gynecology, Ben-Gurion University, Beer Sheba, Israel; ^12^ Department of Obstetrics and Gynecology, Semmelweis University, Budapest, Hungary

**Keywords:** clinical proteomics, diagnostics, high-dimensional, miscarriage, personalized medicine, prevention, reproduction, systems biology

## Abstract

**Introduction:**

Miscarriages affect 50-70% of all gestations and 15-20% of clinically recognized pregnancies. Recurrent pregnancy loss (RPL) occurs in 1-5% of clinical pregnancies and has an enormous demographic impact. However, the etiologies and molecular pathways of RPL are scarcely understood, and therefore, reliable diagnostic and preventive methods are not yet available. Here, we aimed to discover novel biomarkers for RPL using next-generation proteomics technology to help develop early and effective diagnostic tools.

**Methods:**

First-trimester blood samples were collected from women with RPL (*n*=11) and controls with elective termination of pregnancy (*n*=11) between 6–13 weeks of gestation. After immunodepleting 14 highly abundant proteins, plasma samples were reduced, alkylated, and trypsin digested. For the separation of peptides, nano-flow reversed-phase chromatography was applied, and then mass spectrometric analysis was performed. Differentially abundant (DA) proteins were identified using strict criteria and analyzed by protein network and Gene Ontology (GO) enrichment analyses, and two biomarker candidates (CGB and PAPPA) were validated by immunoassay. Biomarker predictive properties were demonstrated using Receiver Operating Characteristic (ROC) curves. Assessments were performed for all cases and then for two gestational age groups, before and after the start of placental circulation [“early RPL”: gestational weeks (GW) 6–9, “late RPL”: GW 9**–**13].

**Results:**

Altogether, 651 proteins were identified and quantified across all samples. When comparing “early control” and “late control” samples, 60 proteins [11 predominantly placenta-expressed (PPE)] were DA. When analyzing all cases, 50 DA proteins were found in RPL (top 3 down: PZP, PSG9, CGB; top 3 up: C4BPA, HBA, HBB), among which 11 PPE proteins were found, all downregulated. Enriched GO terms included ‘placental function’, ‘oxidative processes’, ‘immune function’, and ‘blood coagulation’ related biological processes. When cases were split into early and late RPL groups, 40 DA proteins were identified in early RPL (top 3 down: SHBG, CGB, CGA; top 3 up: C4BPA, SAMP, C4BPB) and 90 in late RPL (top 3 down: PZP, PAPPA, PSG9; top 3 up: THBS1, ECM1, HBB), among which only 15 were shared by both RPL groups. In early RPL, only ‘placental function’ and ‘immune function’ related biological processes were enriched, while in late RPL the top enriched GO terms included ‘placental function’, ‘oxidative processes’, ‘immune function’, ‘blood coagulation’, ‘angiogenesis’, ‘cell migration’, and ‘blood circulation’ related biological processes. Among GO terms, only ‘placental function’ related biological processes were enriched when early- and late RPL DA proteins were analyzed together. Furthermore, the areas under the ROC curves were >0.9 for two protein candidates in all RPL, for five proteins in early RPL, and for ten proteins in late RPL. Among these candidates, CGB and PAPPA were validated by immunoassay which showed a good correlation with MS data (R_CGB_=0.795 and R_PAPPA_=0.965).

**Conclusion:**

We discovered distinct as well as shared molecular pathways associated with RPL pathogenesis before and after the start of placental circulation and identified novel biomarkers for these pathways which have outstanding discriminative properties. Our results may facilitate a better understanding of the molecular pathways of RPL. However, larger clinical studies are needed to investigate whether the identified biomarkers also have predictive power for RPL before pregnancies fail and to test drugs for the modulation of the identified disease pathways and the prevention of RPL. Our findings highlight the importance of the maternal immune system in maintaining successful pregnancy and suggest that targeting immune pathways may offer novel therapeutic approaches for RPL.

## Introduction

Miscarriage is the loss of pregnancy before the 20^th^ week of gestation (1, 2), which affects 50-70% of all conceptions and 15-20% of clinically recognized pregnancies (3–15). Recurrent pregnancy loss (RPL) occurs in 1-5% of clinical pregnancies (6, 10, 16–18) and was defined as the loss of three or more consecutive pregnancies before the 20^th^ week of gestation by the World Health Organization (6, 10, 18–20), and later as two or more failed clinical pregnancies by the American Society for Reproductive Medicine (18, 21). The risk of pregnancy loss during a given gestation was found to increase directly with the number of previous miscarriages (14, 15, 22), however, it is almost the same (i.e. 30% and 33%) after two or three previous miscarriages (17). Therefore, there is an increasing application of the second definition of RPL. In general, miscarriages and RPL have substantial social, psychological, and economic impacts, and in developed countries, where reproductive rates are declining, they may largely influence the demographic processes as well (23, 24).

There are several etiological factors associated with RPL, such as genetic (6, 19, 25–30), anatomical (6, 28, 31–33), immunological (6, 34–40), endocrine (6, 19, 41–45), and thrombotic (6, 46–49) predispositions, including antiphospholipid syndrome (6, 19, 50–53). Moreover, maternal age is also a strong risk factor for subsequent miscarriages (10). Nevertheless, the causes and molecular pathology of about half of the cases are still unknown (6, 13, 18, 21, 54). It is highly probable that causes of RPL vary among different gestational ages, and the timing of miscarriage may have prognostic importance for subsequent pregnancies in each individual case (55–58). However, exact clinical data about gestational ages of miscarriages are not published appropriately in many cases. Therefore, in the future special attention should be paid to the gestational age-specific etiology of RPL. Since the etiologies, molecular pathways, and timing of RPL are not yet comprehensively understood, early and reliable diagnostic and preventive methods are not yet available either.

Pregnancy is a unique immunological state where the maternal immune system must provide tolerance towards the semi-allogenic fetus while maintaining robust defense mechanisms against infections. This complex interaction is orchestrated through immune-modulating mechanisms involving regulatory T cells, cytokines, and trophoblast-derived signals (59–61). Any dysregulation in this immune response can lead to pregnancy complications, including miscarriage and RPL. Altered natural killer cell activity and cytokine profiles can impair maternal-fetal tolerance, leading to placental dysfunction and pregnancy loss (62). Additionally, a heightened inflammatory response, characterized by increased levels of pro-inflammatory cytokines, can contribute to tissue damage and fetal demise (63).

There are several protein biomarker studies in the literature for RPL, either for risk assessment of non-pregnant women (44, 64–70) or for the prediction of the outcome of the current pregnancy (38, 71–84). However, the results of the different studies are often confusing and not comparable because of the heterogeneous patient groups, inadequate definitions, or inappropriate methodology applied (85, 86). Therefore, there is currently no unified protocol for the diagnosis and prediction of RPL.

Although early pregnancy loss is typically defined clinically as a loss before the 13^th^ completed week of gestation (87), in this study we applied a gestational age cut-off at the 9^th^ gestational week to distinguish biologically distinct phases of placental development. Previously, we studied three serum proteins in RPL and defined their exact predictive values (88). Free β-human chorionic gonadotropin (CGB) and pregnancy-associated plasma protein A (PAPPA) were found to be valuable biomarkers for early RPL cases [gestational weeks (GW) 6**–**9], and their discriminative power was excellent for late RPL cases (GW 9**–**13), while placental growth factor (PGF) was a good biomarker for late RPL. The more effective diagnosis of early RPL, as well as a better understanding of the pathology of RPL, requires comprehensive molecular studies on the level of the genome, transcriptome, proteome, and metabolome using state-of-the-art techniques and systems biology approaches. Therefore, here we aimed to discover novel biomarkers for RPL using next-generation proteomics technology and proteome-wide analysis of blood plasma samples collected in a case-control study design including women with RPL and those women undergoing elective termination of pregnancy as a gestational age-matched control group. We used strict clinical definitions and homogenous patient groups. Moreover, standardized sample collection, sample storage, treatment, and analysis, as well as data evaluation using different bioinformatics tools and statistical methods were applied.

## Materials and methods

### Study groups, clinical definitions, and sample collection

The following clinical groups were studied: 1) women with RPL (*n*=11) and control women with elective termination of pregnancy (*n*=11). Each RPL sample was paired with gestational age-matched control. Blood samples were collected within the HUN-PER study at the Maternity Private Clinic of Obstetrics and Gynecology (Budapest, Hungary) at the time of surgery. All women were Caucasian. Samples from pregnancies with congenital or chromosomal abnormalities or multiple gestations were not collected. Pregnancies were dated according to ultrasound scans and samples were collected between 6–13 weeks of gestation. **Table 1** contains the demographic and clinical data of the two study groups.

**Table 1:** Demographic and clinical data of the study groups.

	Proteomics		Immunoassay	
Groups	RPL	Control	RPL	Control
Number of cases^a^	11	11	14	30
Maternal age (years)^a^	36.64 ± 4.84^*^	31.45 ± 6.85	37.21 ± 4.51^***^	30.13 ± 7.02
Gestational age at surgery (GW)^a^	8.99 ± 1.96	8.78 ± 1.96	9.17 ± 1.86	8.61 ± 1.77
Gravidity^b^	3 (2-4)	2 (2-4)	3 (2.25-3.75)^*^	2 (1-3)
Parity^b^	1 (0-1)	1 (0-2)	1 (0-1)	0 (0-1)
Number of miscarriages^b^	2 (2-3)^***^	0 (0-0)	2 (2-2.75)^***^	0 (0-0)

All women were Caucasian.

a Values are presented as numbers (standard deviation (SD)).

b Values are presented as medians (interquartile range (IQR)).

^*^p<0.05 and ^***^p<0.001 compared to controls.

RPL, recurrent pregnancy loss

GW, gestational weeks

RPL was defined as two or more failed clinical pregnancies by the American Society for Reproductive Medicine (21). RPL cases were recruited from patients with a nonviable intrauterine pregnancy detected by ultrasound [gestational sac containing an embryo or fetus without fetal heart activity within the first 12 6/7 weeks of gestation according to the American College of Obstetricians and Gynecologists Practice Bulletin (87)]. We did not pay special concern whether previous failed first-trimester clinical pregnancies were complete/incomplete spontaneous or missed abortions. In the control group, elective termination of pregnancy was performed at the request of pregnant women for non-medical reasons in all cases.

### Processing of blood samples

Blood samples were processed immediately after collection. Plasma and serum samples were separated and stored at −80°C in our Perinatal Biobank at the HUN-REN Research Centre for Natural Sciences.

### Sample preparation for proteomics

Plasma samples were depleted using a Human-14 Multi Affinity Removal Spin Cartridge (Agilent Technologies) according to the manufacturer’s instructions. After the addition of Biognosys’ Lysis Buffer, digestion was carried out on single filter units (Sartorius Vivacon 500, 30’000 MWCO HY) following a modified FASP protocol (described by the Max Planck Institute of Biochemistry, Martinsried, Germany).

Samples were reduced and alkylated using Biognosys’ Reduction & Alkylation Solution for 30min at 37°C. Subsequently, digestion to peptides was carried out using trypsin (Promega Corporation, Madison, WI, USA) overnight at 37°C and a protein:protease ratio of 1:100.

### Clean-up for mass spectrometry

Peptides were desalted using C18 Midi BioPure columns (NestGroup, Ipswich, MA, USA) according to the manufacturer’s instructions and dried down using a SpeedVac system. Peptides were resuspended in LC solvent A [1% acetonitrile, 0.1% formic acid (FA)] (Sigma-Aldrich, St. Louis, MO, USA). Prior to mass spectrometric analyses, they were spiked with Biognosys’ iRT kit calibration peptides. Peptide concentrations were determined using a UV/VIS Spectrometer (SPECTROstar Nano, BMG Labtech Ortenberg, Germany).

### High-Resolution Mass Spectrometry using Tandem Mass Spectrometry

For High-Resolution Mass Liquid Chromatography-Tandem Mass Spectrometry (HRM LC-MS/MS) measurements, 2µg of peptides were injected into an in-house packed C18 column (Dr. Maisch ReproSil Pur, 1.9µm particle size, 120Å pore size; 75µm inner diameter, 50cm length, New Objective) on a Thermo Scientific™ Easy nLC 1200 nano-liquid chromatography system connected to a Thermo Scientific™ Q Exactive™ HF-X mass spectrometer equipped with a standard nano-electrospray source. LC solvents were A: 1% acetonitrile in water with 0.1% FA; B: 15% water in acetonitrile with 0.1% FA (all solvents from Sigma-Aldrich, St. Louis, MO, USA). The nonlinear LC gradient was 1-52% solvent B in 60min followed by 52-90% B in 10sec, 90% B for 10min, 90-1% B in 10sec and 1% B for 5min. A data-independent acquisition (DIA) method with one full-range survey scan and 14 DIA windows was used.

### Immunoassay

Three selected biomarker proteins were analyzed applying the same study groups (RPL, *n*=14; control, *n*=30) including the same samples as described in our previous study (88). Briefly, serum concentrations of CGB, PAPPA, and PGF proteins were measured with immunoassay, using a BRAHMS plus KRYPTOR Analyzer (ThermoFisher Scientific, Waltham, MA, USA) with monoclonal antibodies targeted to different epitopes of the measured analytes. Here, the results of CGB and PAPPA were used for re-analysis.

### Data analysis

#### Analysis of HRM mass spectrometric data

HRM mass spectrometric data were analyzed using Spectronaut™ X software (Biognosys). The false discovery rate (FDR) on peptide and protein levels was set to 1%, data was filtered using row-based extraction. An assay library (protein inventory) generated previously (combined from plasma and placenta samples) was used for the analysis, and depleted proteins were removed from the dataset. The HRM measurements analyzed with Spectronaut were normalized using local regression normalization, and sparse protein profiles were used for the normalization of the samples (89). Sparse filtered data (no missing values, identification was included if the target was identified in at least one sample with *q*-value <0.01) were reported. Protein quantities were determined by averaging the intensities of the top 1–3 peptides, ranked by Spectronaut.

#### Identification of placenta-specific proteins

In this study, we also focused on predominantly placenta-expressed (PPE) proteins, as they serve as molecular markers of placental development and function. PPE proteins were specified as proteins encoded by PPE genes (n=164) as defined by Than et al. (90) and Szilagyi et al. (91). PPE genes’ placental expression is high which is unique or several folds higher than in other tissues, and their dysregulation may reflect altered placental functions.

#### Identification of differentially abundant proteins

A two-sample unpaired Student’s *t*-test was performed for the protein intensities between RPL and control samples. The protein regulation was calculated as the log_2_ ratios of the individual protein intensities averaged over the study groups. *P*-values were corrected for multiple testing using the *q*-value approach (92) to control the overall false discovery rate. Up- and down-regulated proteins were selected by two filtering cut-offs: *q*-values <0.1 and absolute log_2_ (fold-change) >log_2_(1.5).

We split samples into two groups according to the gestational age at the time of sampling: one for samples with gestational age <9 weeks (*n*=7) and another for samples taken at gestational age ≥9 weeks (*n*=4). DA proteins were calculated, and further analyses were performed also for early RPL and late RPL separately. This cut-off was selected based on the culmination of two critical developmental milestones, the luteal-placental shift and the onset of intervillous circulation. The luteal-placental shift in which progesterone production transitions from the corpus luteum to the placenta typically occurs gradually between the 6th to 9th week of gestation as shown first by the pioneering study of Csapo et al. (58, 93, 94). In addition, trophoblastic plugs that provide hypoxic environment for the developing embryo at the beginning of pregnancy start to dissolve at around 9–10 weeks of gestation, giving place to the start of the maternal circulation of the placenta as evidenced by the rising oxygen tension and change in flow patterns detected with Doppler ultrasound. This is also the time period when the first wave of extravillous trophoblast invasion starts to occur, radically altering the placental milieu (95). Therefore, using 9 weeks as a biological cut-off provides a meaningful demarcation for analyzing gestational age–dependent molecular changes in maternal plasma.

#### Protein network and Gene Ontology enrichment analyses

Protein networks were retrieved from the STRING database (96) for proteins in the sparse filtered dataset and the following thresholds for candidate identification: *q*-value <0.1, absolute log_2_ fold-change >1.5. Gene Ontology (GO) (97) enrichment analysis was performed using the STRING website, and biological processes were checked for over-representation within the set of candidates.

Results are presented using volcano plots showing *q*-values and fold changes for down- and upregulated DA as well as non-DA proteins, and mosaic plots showing the enrichment of PPE proteins among different groups. We also present the interaction networks of DA proteins retrieved from the STRING database, and the most enriched GO biological processes in the various subgroups.

#### Classifier to discriminate between RPL and control groups

We trained a Logistic Regression (LR) classifier for each DA protein separately to estimate the predictive power of single proteins. The training process was preceded by hyperparameter optimization. The solver and regularization methods obtained by the optimization process were used for the classifier. An AUC value averaging over 50 runs of five-fold cross-validation procedures was calculated.

To account for potential differences in classification performance at different stages of pregnancy, we trained gestational age-specific classifiers using the same evaluation procedure. Separate classifiers were trained for the two groups of samples, employing an LR classifier for each DA protein. The models were assessed using 50 runs of three-fold cross-validation, and the resulting AUC values were averaged to evaluate predictive performance.

#### Immunoassay re-analysis and correlation analysis

To validate the proteomic results, we re-analyzed published immunoassay data (88) on the abundance of two clinically established biomarkers **–** PAPPA and CGB **–** in a set of plasma samples (total *n*=44). This included 22 samples that overlapped with the current mass spectrometry cohort, and an additional 22 samples that were independent of this study.

Former immunoassay results of CGB and PAPPA (88) were re-analyzed for the overlapping cohort. Correlation analysis was performed between the immunoassay and proteomic results, and the Pearson correlation coefficients (R) were calculated.

## Results

### Overall demographic, clinical and proteomic characteristics of the study population

Demographic and clinical information for both the proteomic and validation cohorts is summarized in **Table 1**. In both sets, the mean maternal age was higher in the RPL than in the control group. Women in the RPL group had experienced two to four spontaneous miscarriages, including the current one, while those in the control group did not have any previous miscarriages.

Using high-resolution mass spectrometry with a strict limit of quantification, we identified and quantified 651 proteins across all plasma samples (RPL, *n*=11; control, *n*=11), of which 27 were classified as PPE proteins (**Supplementary Table 1**).

Given the higher average maternal age in the RPL group, we investigated whether maternal age might act as a potential confounding factor influencing the proteomic data. To assess this, we performed a correlation analysis between maternal age and the abundance of all quantified proteins across the entire cohort. After correcting for multiple hypothesis testing using Storey’s *q*-value method, none of the proteins showed a statistically significant association with maternal age, suggesting that maternal age did not have a detectable impact on the observed proteomic differences.

### Changes in the maternal plasma proteome in the first trimester in control pregnancies

**Figure 1:**
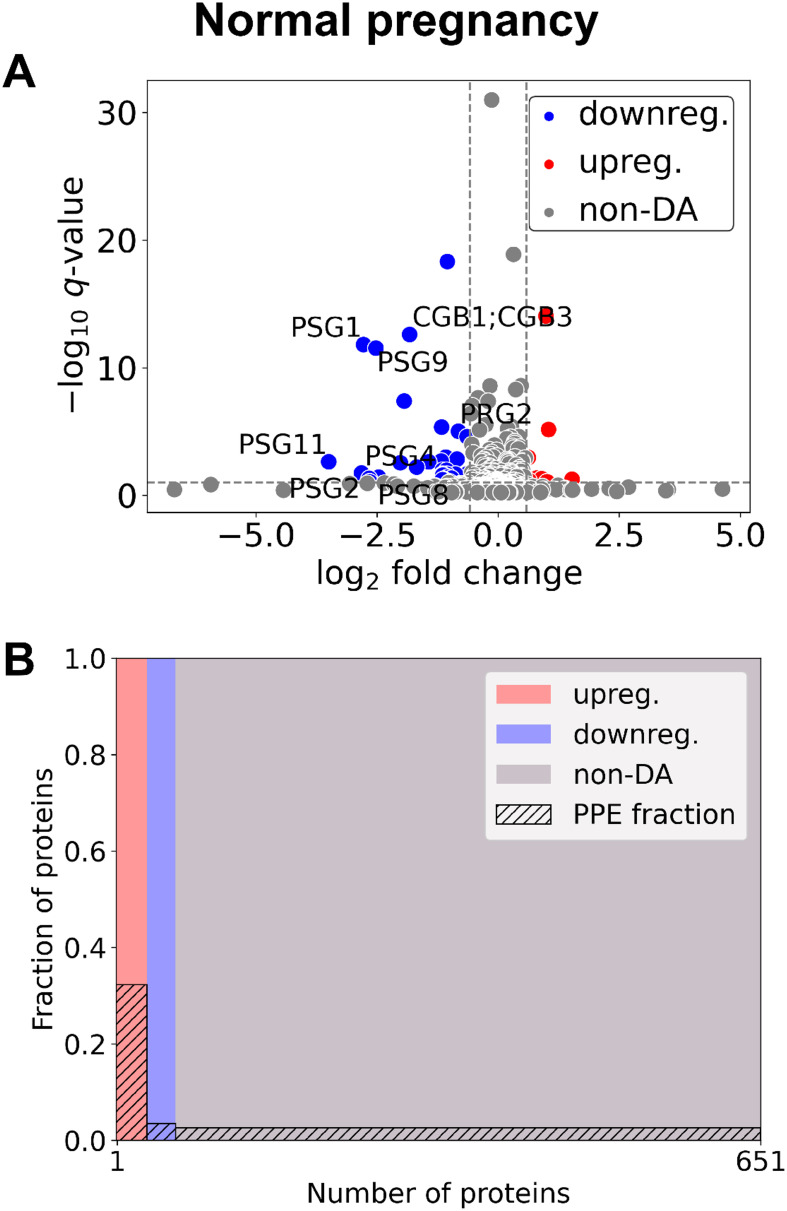
Changes of the maternal plasma proteome in the first trimester of normal pregnancies. **(A)** Changes in protein intensities among late- (GW 9**–**13) and early control samples (GW 6**–**9) are represented using a volcano plot. Proteins are shown in terms of their measured differences in abundance (x-axis) and the significance of the difference (y-axis). Differences in abundance are represented as log_2_(fold change). The significance is represented as a –log_10_ (*q*-value). Dotted lines represent the thresholds used to select the probes for DA proteins: absolute log_2_ fold change 1.5 and –log_10_ (*q*-value) >1 for statistical significance. Downregulated proteins are shown in blue, upregulated proteins are marked in red, and non-DA proteins are shown as grey dots. Gene IDs of PPE DA proteins are shown as well. **(B)** Mosaic plot shows the enrichment of PPE proteins (striped part). The width of each colored rectangle is proportional to the number of proteins in the corresponding group, and the height of the striped part is proportional to the fraction of PPE proteins within the particular group. Upregulated proteins are marked red, downregulated proteins blue, and non-DA proteins grey. DA, differentially abundant; GW, gestational weeks; PPE, predominantly placenta-expressed.

Between early control (*n*=7) and late control (*n*=4) samples (**Figure 1**, **Supplementary Table 2**), 60 DA proteins were identified, among them 11 were PPE proteins (enrichment factor: 4.59, *p*=4.31×10^-6^). 29 proteins were downregulated (one PPE, enrichment non-significant) and 31 upregulated (ten PPE, enrichment factor: 8.08, *p*=4.11×10^-8^).

### Changes in the maternal plasma proteome in the first trimester in RPL

**Figure 2:**
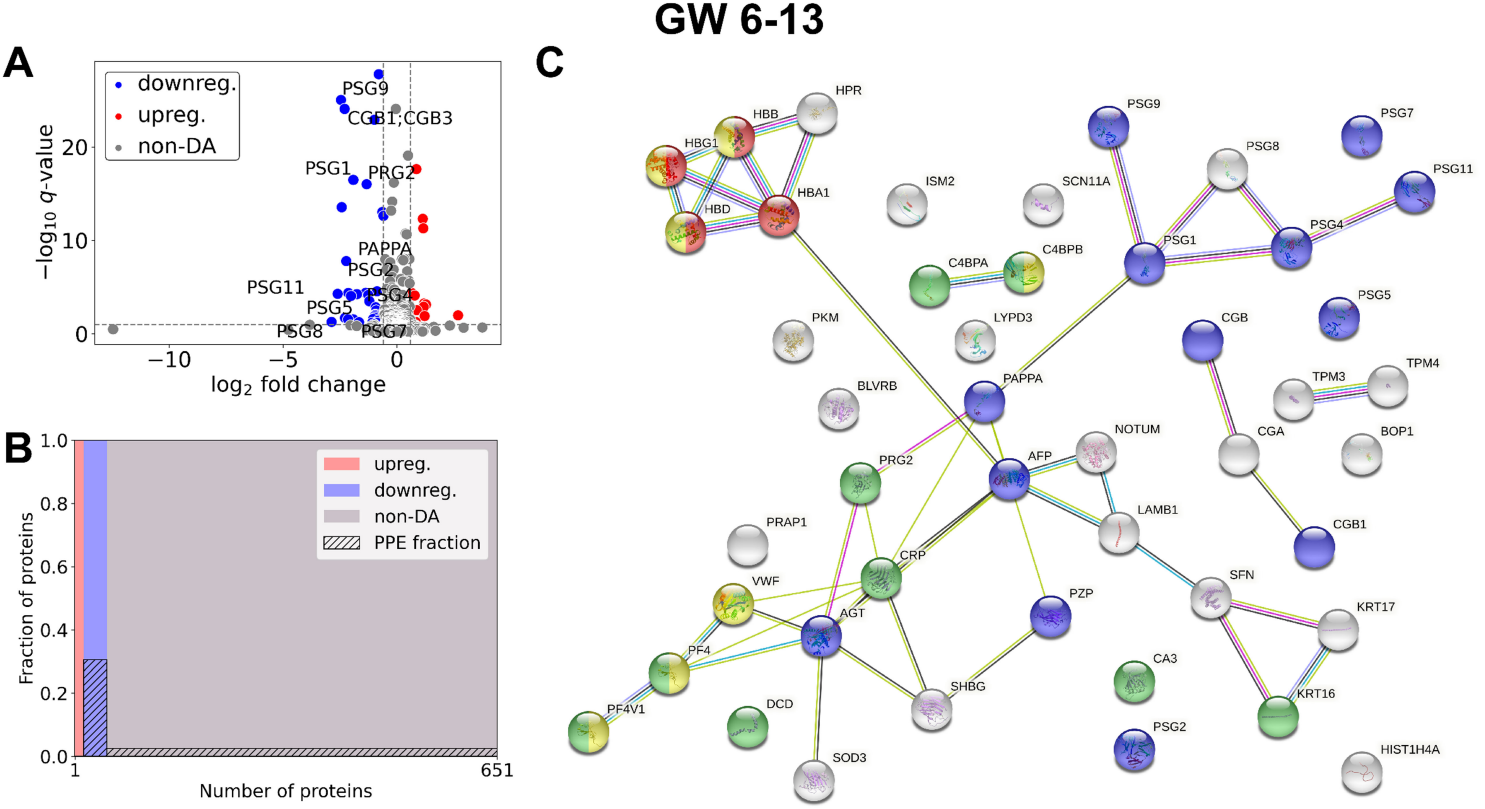
Comparison of RPL and control samples in the whole gestational age range. **(A)** Changes in protein intensities among RPL (GW 6**–**13) and control samples (GW 6**–**13) are represented using a volcano plot. Downregulated proteins are shown in blue, upregulated proteins are marked in red, and non-DA proteins are shown as grey dots. Gene IDs of PPE DA proteins are shown as well. **(B)** Mosaic plot shows the enrichment of PPE proteins (striped part). Upregulated proteins are marked red, downregulated proteins blue, and non-DA proteins grey. **(C)** The interaction network of DA proteins and GO biological process analysis was performed using STRING. On the figure components of selected GO terms are marked with different colors: ‘multi-organism reproductive process’ (blue), components of ‘oxygen transport’ (red), ‘response to other organism’ (green), and ‘blood coagulation’ (yellow). DA, differentially abundant; GO, Gene Ontology; GW, gestational weeks; PPE, predominantly placenta-expressed; RPL, recurrent pregnancy loss.

In the whole gestational age range (**Figure 2**, **Table 2**, **Supplementary Tables 2, 3**) 50 DA proteins were identified (top 3 down: PZP, PSG9, CGB; top 3 up: C4BPA, HBA, HBB), 36 were downregulated and 14 were upregulated. Eleven PPE proteins were found among DA proteins, corresponding to an enrichment factor of 5.51 and *p*=5.91×10^-7^. All of these PPE proteins were down-regulated in RPL (enrichment factor: 7.65, *p*=1.31×10^-8^). Significantly enriched GO terms included ‘placental function’ (3/27), ‘oxidative processes’ (4/27), ‘immune function’ (8/27), and ‘blood coagulation’ (1/27) related biological processes.

**Table 2:** Top DA proteins in RPL during GW 6-13.

Uniprot ID	Gene Symbol	Protein name	Log_2_ ratio	*P* value	*Q* value
P20742	*PZP*	Pregnancy zone protein	-0.80	8.28×10^-31^	1.59×10^-28^
Q00887	*PSG9*	Pregnancy-specific beta-1-glycoprotein 9	-2.46	7.3×10^-28^	9.348×10^-26^
A6NKQ9;P0DN86	*CGB1;CGB3*	Choriogonadotropin subunit beta variant 1;Choriogonadotropin subunit beta 3	-2.30	1.09×10^-26^	8.39×10^-25^
P04278	*SHBG*	Sex hormone-binding globulin	-0.99	1.87×10^-25^	1.195×10^-23^
P04003	*C4BPA*	C4b-binding protein alpha chain	0.85	4.77×10^-20^	2.288×10^-18^

**Figure 3:**
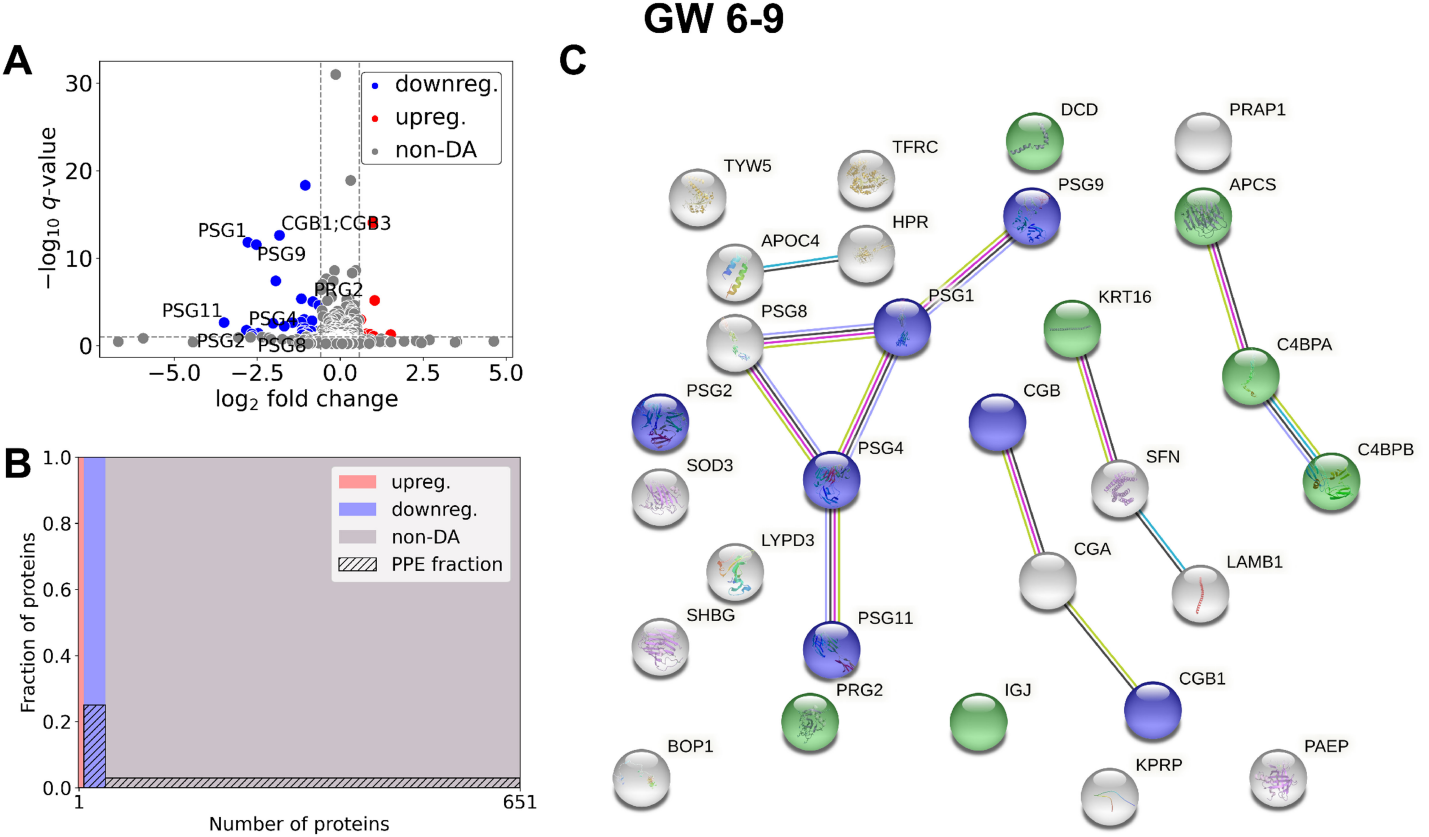
Comparison of RPL and control samples in the early period. **(A)** Changes in protein intensities among early RPL (GW 6**–**9) and early control samples (GW 6**–**9) are represented using a volcano plot. Downregulated proteins are shown in blue, upregulated proteins are marked in red, and non-DA proteins are shown as grey dots. Gene IDs of PPE DA proteins are shown as well. **(B)** Mosaic plot shows the enrichment of PPE proteins (striped part). Upregulated proteins are marked red, downregulated proteins blue, and non-DA proteins grey. **(C)** On the interaction networks of DA proteins components of selected GO terms are marked with different colors: ‘multi-organism reproductive process’ (blue) and ‘defense response to other organism’ (green). DA, differentially abundant; GO, Gene Ontology; GW, gestational weeks; PPE, predominantly placenta-expressed; RPL, recurrent pregnancy loss.

In early RPL (**Figure 3**, **Table 3**, **Supplementary Tables 2, 3**), 40 DA proteins were identified (top 3 down: SHBG, CGB, CGA; top 3 up: C4BPA, SAMP, C4BPB), 32 were downregulated and 8 were upregulated. Among these, eight PPE proteins were found (enrichment factor: 5.01, *p*=6.91×10^-5^). In early RPL, also all PPE proteins were downregulated (enrichment factor: 6.26, *p*=1.16×10^-5^) and the fold changes of these proteins were also the lowest with one exception. Placental (3/14), as well as immune function (7/14) -related biological processes were among the top enriched GO terms.

**Table 3:** Top DA proteins in RPL during GW 6-9.

Uniprot ID	Gene Symbol	Protein name	Log_2_ ratio	*P* value	*Q* value
P04278	*SHBG*	Sex hormone-binding globulin	-1.05	6.96×10^-21^	4.861×10^-19^
P04003	*C4BPA*	C4b-binding protein alpha chain	0.988	1.96×10^-16^	8.968×10^-15^
A6NKQ9; P0DN86	*CGB1; CGB3*	Choriogonadotropin subunit beta variant 1; Choriogonadotropin subunit beta 3	-1.83	6.7×10^-15^	2.486×10^-13^
P11464	*PSG1*	Pregnancy-specific beta-1-glycoprotein 1	-2.78	4.7×10^-14^	1.55×10^-12^
Q00887	*PSG9*	Pregnancy-specific beta-1-glycoprotein 9	-2.52	9.53×10^-14^	2.902×10^-12^

**Figure 4:**
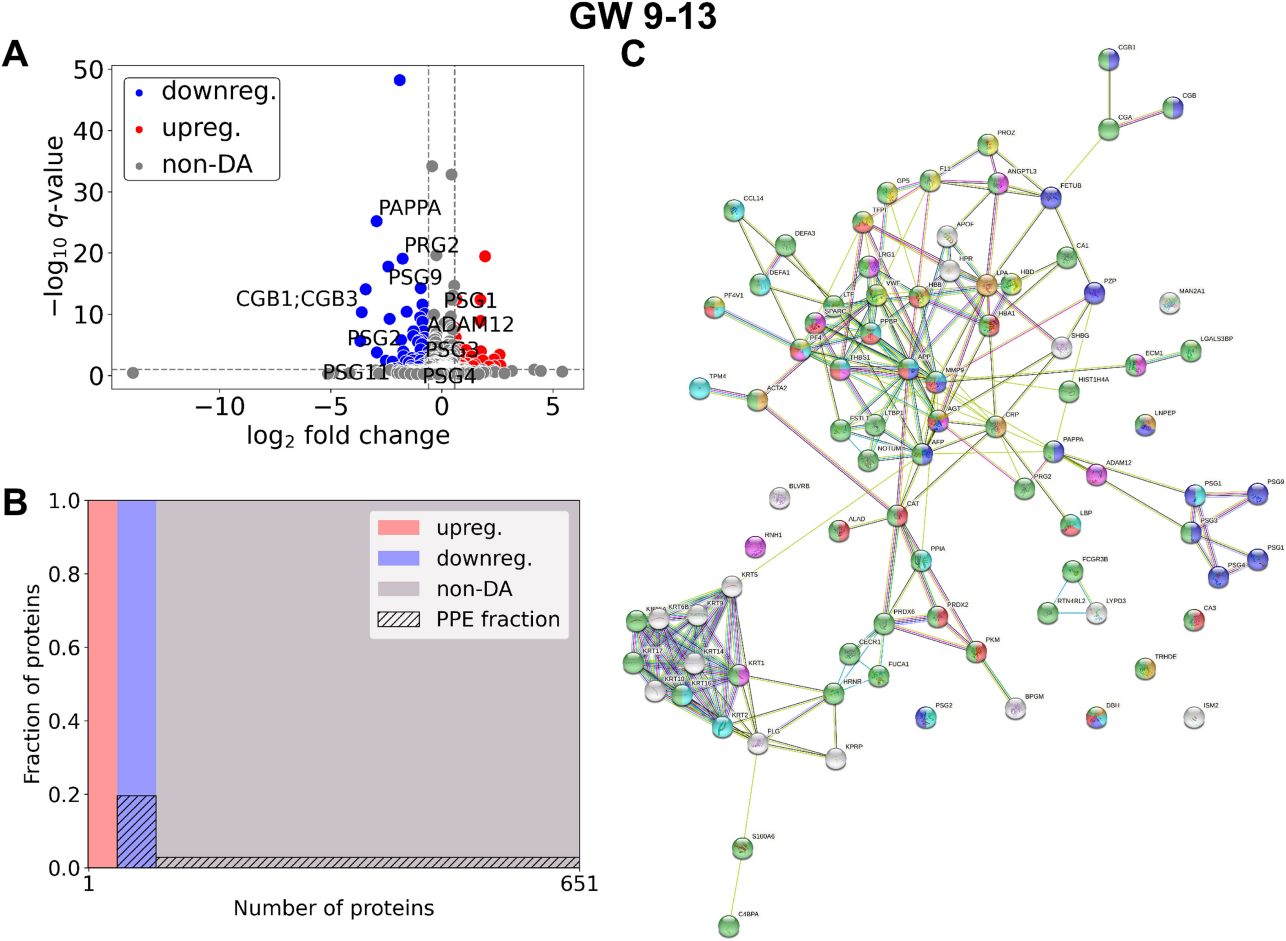
Comparison of RPL and control samples in the late period. **(A)** Changes in protein intensities among late RPL (GW 9**–**13) and late control samples (GW 9**–**13) are represented using a volcano plot. Downregulated proteins are shown in blue, upregulated proteins are marked in red, and non-DA proteins are shown as grey dots. The Gene ID of PPE DA proteins is shown as well. **(B)** Mosaic plot shows the enrichment of PPE proteins (striped part). Upregulated proteins are marked red, downregulated proteins blue, and non-DA proteins grey. **(C)** On the interaction networks of DA proteins components of selected GO terms are marked with different colors: ‘reproductive process’ (blue), components of ‘response to oxygen-containing compound’ (red), ‘response to stimulus’ (green), ‘blood coagulation’ (yellow), ‘regulation of angiogenesis’ (purple), ‘movement of cell or subcellular component’ (light blue), and ‘blood circulation’ (orange). DA, differentially abundant; GO, Gene Ontology; GW, gestational weeks; PPE, predominantly placenta-expressed; RPL, recurrent pregnancy loss.

In late RPL (**Figure 4**, **Table 4**, **Supplementary Tables 2, 3**) 90 DA proteins were found (top 3 down: PZP, PAPPA, PSG9; top 3 up: THBS1, ECM, HBB), among them 51 were downregulated and 39 were upregulated. Ten PPE proteins were found (enrichment factor: 2.78, *p*=1.25×10^-3^). In late RPL, similar to early RPL, all of the PPE proteins were downregulated (enrichment factor: 4.91, *p*=7.36×10^-6^), and the fold changes of these proteins were also among the lowest ones with two exceptions. The top enriched GO terms included ‘placental function’ (4/206), ‘oxidative processes’ (8/206), ‘immune function’ (55/206), ‘blood coagulation’ (6/206), ‘angiogenesis’ (3/206), ‘cell migration’ (9/206), as well as ‘blood circulation’ (6/206) -related biological processes.

**Table 4:** Top DA proteins in RPL during GW 9-13.

Uniprot ID	Gene Symbol	Protein name	Log_2_ ratio	*P* value	*Q* value
P20742	*PZP*	Pregnancy zone protein	-1.89	1.06×10^-51^	6.31×10^-49^
Q13219	*PAPPA*	Pappalysin-1	-2.93	4.19×10^-28^	6.64×10^-26^
P07996	*THBS1*	Thrombospondin-1	1.96	4.09×10^-22^	3.60×10^-20^
P13727	*PRG2*	Bone marrow proteoglycan	-1.76	1.03×10^-21^	8.43×10^-20^
Q00887	*PSG9*	Pregnancy-specific beta-1-glycoprotein 9	-2.41	2.65×10^-20^	1.70×10^-18^

**Figure 5:**
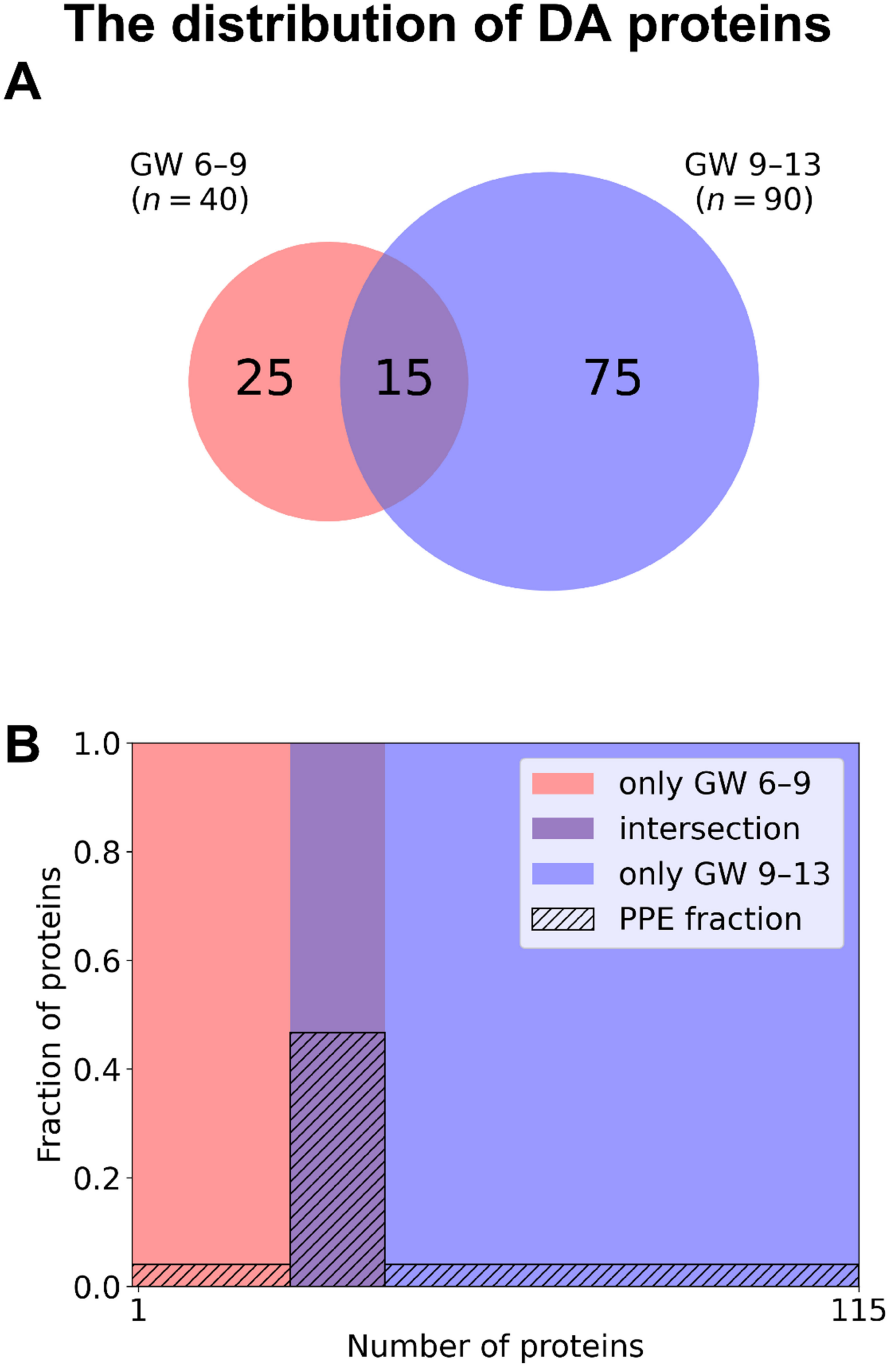
The distribution of DA proteins among early and late samples. **(A)** The number of DA proteins is displayed on a Venn diagram for early RPL (red, GW 6**–**9) and for late RPL (blue, GW 9**–**13) separately, as well as for both sub-groups (purple). **(B)** Mosaic plot shows the enrichment of PPE proteins (striped part). DA proteins only in early RPL are shown in red, DA proteins only in late RPL in blue, and common DA proteins in purple. DA, differentially abundant; GW, gestational weeks; PPE, predominantly placenta-expressed; RPL, recurrent pregnancy loss.

Among DA proteins (**Figure 5**, **Supplementary Tables 2, 3**), 15 were identified in both early and late RPL, including seven PPE proteins (enrichment factor: 4.88, *p*=3.70×10^-5^). 25 proteins (one PPE, non-significant) were found only in early RPL, and 75 (three PPE, non-significant) only in late RPL. Importantly, among DA proteins identified in both early and late RPL, only ‘placental function’-related biological processes (3/4) were enriched.

### The discriminative power of plasma proteome and DA proteins with respect to study groups

Principal Component Analysis (PCA) was performed to identify the variance of data in the different sample groups (RPL and control groups) based on all proteins. Principal components 1, 2, and 3 explained 31.7% of the total variance, and little separation between sample groups could be observed (data not shown).

**Figure 6:**
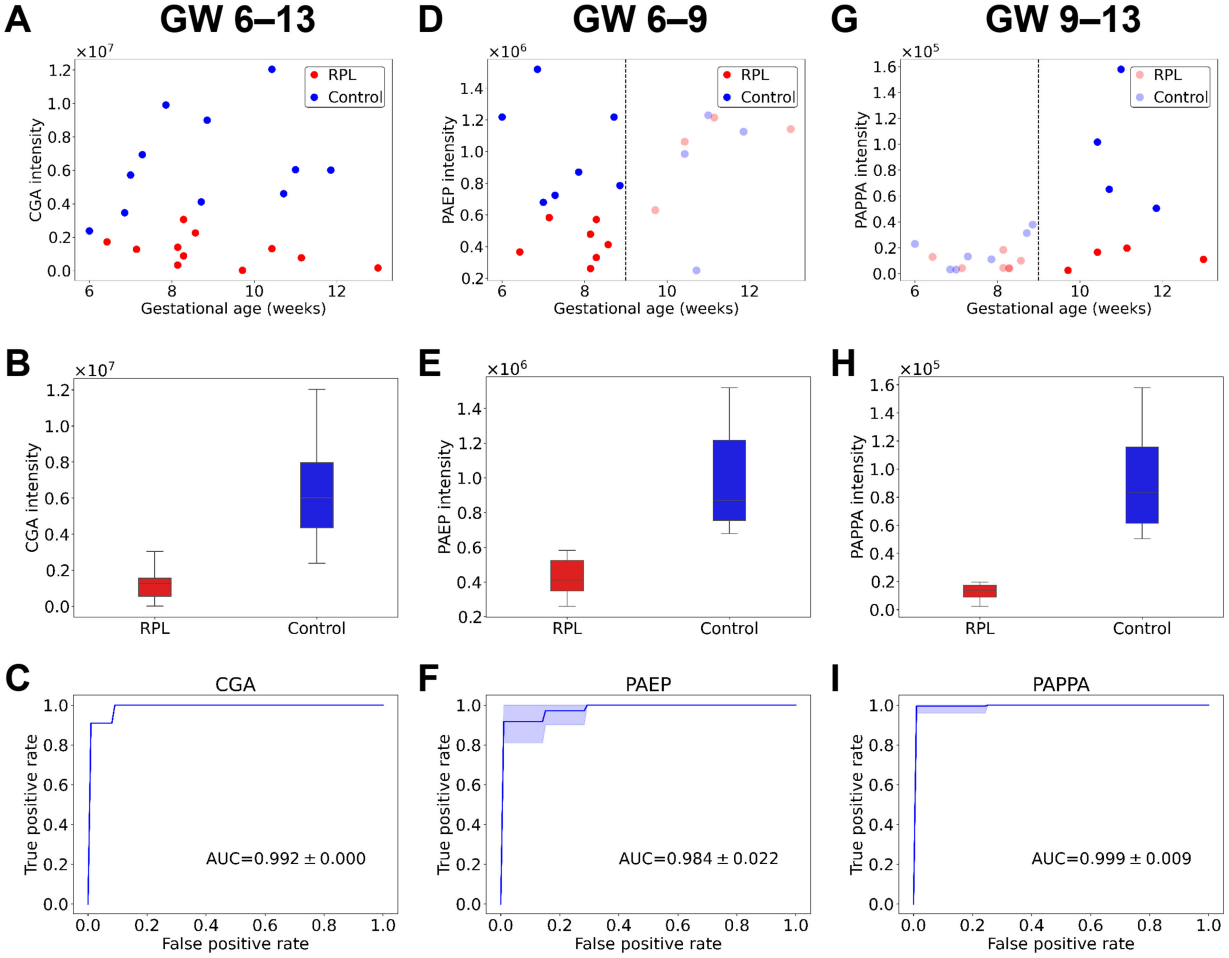
Protein intensities and classification performances of the best biomarker candidates for RPL. Scatter plots show protein intensities *vs*. gestational age for the best biomarker candidates in the whole gestational age range (**A**, GW 6**–**13), for early RPL (**D,** GW 6**–**9), and late RPL (**G**, GW 9**–**13). Box plots represent the intensities of the best biomarker candidates in the whole gestational age range **(B)**, for early RPL **(E)**, and late RPL **(H)**. ROC curves represent classification performances of the best biomarker candidates in the whole gestational age range **(C)**, for early RPL **(F)**, and late RPL **(I)**. On the plots, the RPL group is marked with red and the control group with blue. AUC, area under the curve; GW, gestational weeks; ROC, receiver operating characteristic; RPL, recurrent pregnancy loss.

Classification performances were characterized by calculating sensitivity, specificity, and ROC curves for each DA protein in the different gestational age ranges. Scatter plots, box plots, and ROC curves of the best candidate proteins (whole gestational age range: CGA, early RPL: PAEP, late RPL: PAPPA) are presented in **Figure 6**, respectively.

### Validation of mass spectrometry results with immunoassays

**Figure 7:**
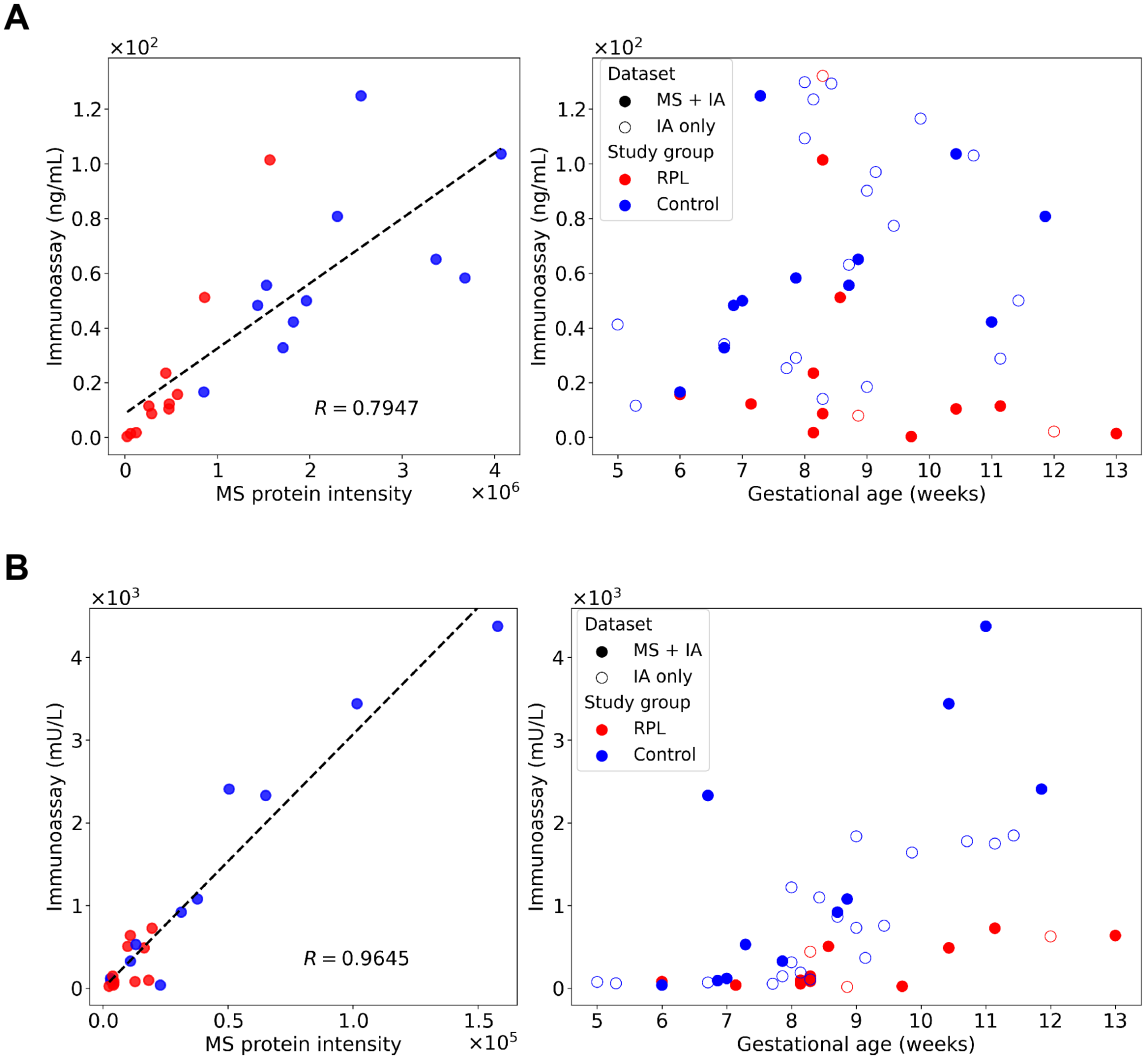
Correlation analysis between immunoassay and mass spectrometry (MS) results and validation of proteomic findings. Concentration of CGB **(A)** and PAPPA **(B)** were determined using immunoassay in our previous study (88) and re-evaluated to validate the proteomic results. Correlation between the immunoassay concentration and MS relative intensity results were calculated for both proteins for the identical samples (RPL, *n*=11; and control group, *n*=11). Pearson correlation coefficients (R) were 0.7947 for CGB and 0.9645 for PAPPA, respectively. Immunoassay concentrations were plotted against gestational age, with overlapping and distinct samples are denoted with different symbols. CGB, β-human chorionic gonadotropin; MS, mass spectrometry; R, Pearson correlation coefficient; PAPPA, pregnancy-associated plasma protein A; RPL, recurrent pregnancy loss.

The concentration of CGB and PAPPA was also determined using the same immunoassay as in our previous study (88). In concordance with the proteomic findings, similar trends in biomarker levels were observed in the validation cohort. Both CGB and PAPPA showed significantly lower concentrations in the RPL group compared to the control group, reinforcing the proteomic results (**Figure 7**).

The correlation analysis between immunoassay and mass spectrometry data revealed a good correlation (**Figure 7**) on the overlapping cohort as the Pearson correlation coefficient (R) was 0.795 for CGB and 0.965 for PAPPA.

## Discussion

### Principal findings of this study

1) Sixty DA proteins had gestational age-related concentration changes in controls. 2) Fifty DA proteins were dysregulated in RPL compared to control samples. 3) Forty DA proteins were found in early RPL and ninety in late RPL. 4) PPE proteins were enriched among DA proteins and showed to be the most downregulated during both periods in RPL. 5) Enriched GO terms in early RPL included ‘immune regulation’, ‘complement activation’, and ‘female pregnancy’ while ‘placental function’, ‘oxidative processes’, ‘immune function’, and ‘blood coagulation’ related biological processes were enriched in late RPL among others. 6) Several biomarker candidates for early and late RPL were identified and two were validated with immunoassay.

### Plasma proteome profiling and analysis of PPE proteins in the maternal blood using mass spectrometry

The fetal support and maintenance of pregnancy by providing nutrition, immunological and hormonal regulation as well as gas and waste exchange, are the most important roles of the placenta (98, 99). It has been known for more than half century that protein products of the placenta get into the maternal circulation, and their circulating concentration changes may well reflect changes in placental functions with gestational age in normal pregnancies as well as dysregulation of placental functions in pregnancy complications. Therefore, placental proteins turned out to be of great significance for the monitoring of placental functions through non-invasive sampling of maternal blood (i.e. liquid biopsy) (100–103).

We have recently defined PPE proteins (90, 91), which play a central role in monitoring placental functions in the maternal blood. Also, our previous results showed that placental dysfunction is often associated with the altered expression of PPE proteins (104–113), therefore, special attention should be paid to these proteins in the prediction and treatment of pregnancy complications such as RPL. Recently, with immunoassay, we examined three of these PPE proteins (free β-hCG, PAPP-A, and PlGF) and confirmed their diagnostic significance in RPL (88). Utilizing the recent developments in the field of proteomics and mass spectrometry that enable the deep proteomic investigation of different tissues (114), including the placenta (115), and the liquid biopsy of these tissues via minimal invasive blood draw (116, 117), here we aimed to explore the proteome-wide changes in maternal plasma in RPL. This current analysis had a special attention to PPE proteins in an attempt to provide relevant information about the developmental status and functional condition of the placenta and to support the early detection and/or prediction of failing placental functions.

### Changing maternal plasma proteomic signature in normal pregnancy during the first trimester

The placenta undergoes rapid development in the first weeks of pregnancy (118), however, the lack of its connection to the maternal circulation may hinder placental liquid biopsy at the beginning of the pregnancy (119, 120). Indeed, villous placental vessels and circulation start to develop at around gestational weeks 5–6 (121, 122), while the intervillous circulation is only being established after around the 9^th^ gestational week. This is a protective mechanism for the developing embryo and placenta to defend their tissues from excessive and harmful oxygen exposure (95, 123). Therefore, placental products enter into the maternal circulation in much larger quantities only after the opening of the intervillous blood space (124).

To account for this physiological change in placental circulation during the first trimester, we investigated the proteomic changes before and after the supposed start of the intervillous circulation while considering differences in maternal environmental, metabolic, and immunological characteristics. In control pregnancies, our study identified key DA proteins between gestational weeks 6–9 and 9–13, shedding light on the physiological adaptations during early pregnancy. There were 29 proteins with elevated concentrations in gestational weeks 6–9 compared to 9–13. These included FN1 (fibronectin 1) (125), ECM1 (extracellular matrix protein 1) (126), and COMP (cartilage oligomeric matrix protein) (127), which are all associated with extracellular matrix organization, cell adhesion, and tissue remodeling. Their dropping concentration with gestational age suggests a shift from initial implantation and rapid tissue expansion toward the established placental milieu. Other proteins with decreasing concentration over gestational age included PF4 (platelet factor 4), which is a key regulator of vascular homeostasis (128, 129), highlighting the high demands for vascular remodeling during the early phase of placentation. Furthermore, IGFBP2 (insulin-like growth factor-binding protein 2), which regulates the bioavailability of insulin-like growth factors crucial for cellular proliferation and differentiation (130), had a decreasing concentration with advancing gestational age. Several platelet-related proteins (e.g., PF4, PF4V1, PPBP alias CXCL7, THBS1, and VWF) were also found to decrease over gestational age. This may be due to a decrease in mean platelet count gradually throughout gestation; otherwise, upon activation, maternal platelets would provide a source of proinflammatory mediators in the intervillous space of the placenta (129).

Conversely, 31 proteins demonstrated increased concentrations in gestational weeks 9–13 compared to 6–9. These included PZP (pregnancy zone protein) which supports the transition towards immune tolerance (131, 132), as well as PAPPA which increases the bioavailability of insulin-like growth factors, which are crucial for fetal growth and placental development. Also, ADAM12 (a disintegrin and metalloproteinase domain-containing protein 12) is a well-known regulator of trophoblast invasion and extracellular matrix (ECM) remodeling (133). Proteins such as PRG2 (proteoglycan 2, pro eosinophil major basic protein), PSG1, PSG3, and PSG9 (pregnancy-specific beta-1 glycoprotein 1, 3, and 9) are key immunomodulators that help to establish maternal immune tolerance towards the fetus (134). Many of these proteins are also well-known to have increasing concentrations in maternal blood during this timeframe of pregnancy which validates our study (112, 116). These findings provide insights into the dynamic proteomic changes occurring in early pregnancy. The elevated concentrations of ECM-related proteins (FN1, ECM1, COMP) and vascular regulators (PF4) in the earlier phase suggest the importance of ECM reorganization, tissue remodeling, and vascular homeostasis essential for implantation and initial placental formation. ECM proteins not only provide structural scaffolding for invading trophoblasts but also participate in immune regulation by modulating leukocyte adhesion and migration. FN1, for instance, can interact with integrins and Toll-like receptors on maternal immune cells, influencing macrophage activation and cytokine secretion (135).

In contrast, the increased abundance of proteins after week 9, involved in immunomodulation (PZP, PSG1, PSG9, PRG2) and placental development (PAPPA, ADAM12), aligns with the physiological progression toward immune tolerance and increased placental growth.

PZP, a pregnancy-associated protease inhibitor, has been shown to suppress T-cell proliferation and promote a tolerogenic environment by forming complexes with glycodelin-A, and leading to reduced IL-2 production (131).

Pregnancy-specific glycoproteins (PSG1, PSG3, PSG9) are produced by the syncytiotrophoblast (136) and act on maternal monocytes, dendritic cells, and NK cells to induce anti-inflammatory cytokines (e.g., IL-10) and activate TGF-β1 and TGF-β2 (134, 137), which together promote the expansion of regulatory T cells (Tregs) and dampen cytotoxic responses (138).

ADAM12 plays a dual role, facilitating trophoblast invasion through ECM remodeling while also influencing the availability of cytokines and growth factors within the decidua hence contributes to the pathogenesis of tissue inflammation and TH1 differentiation (139). Altogether, these proteins help in reprogramming the maternal immune system, essential for maintaining maternal-fetal tolerance as the intervillous circulation opens and the fetal-derived placenta becomes more exposed to maternal immunity.

### Immune dysregulation and impaired maternal-fetal tolerance specific for early RPL

In early RPL cases, we identified the predominant changes in the immune-related proteome. Several proteins involved in immune tolerance and complement regulation were downregulated in early RPL, including C4BPA (complement component 4 binding protein alpha) and C4BPB (complement component 4 binding protein beta), which play crucial roles in modulating complement activation and preventing excessive immune responses at the maternal-fetal interface (140). Additionally, PAEP (progestagen-associated endometrial protein, also known as glycodelin) (141), a critical modulator of maternal immune tolerance that suppresses T-cell activation and supports embryo implantation (142, 143), showed decreased levels in early RPL cases. In addition, GO enrichment analysis further highlighted the disrupted biological processes in early RPL, with enriched terms predominantly linked to ‘immune regulation’, ‘complement activation’, and ‘female pregnancy’. Key terms included ‘multi-organism processes’ (reflecting maternal-fetal interactions), ‘regulation of opsonization’, and ‘negative regulation of complement activation’. These findings may reflect impaired regulation of immune interactions and tolerance mechanisms at the maternal-fetal interface in RPL.

### Placental dysregulation specific for late RPL

The proteomic profile in late RPL revealed an increased number of DA proteins associated with angiogenesis and inflammatory responses. ANGPTL3 (angiopoietin-related protein 3), a regulator of lipid metabolism (144), has been implicated in modulating angiogenesis (145) and may contribute to altered placental vascularization. PF4, a chemokine released from activated platelets, is involved in immune cell recruitment and inflammatory signaling (146, 147), and its elevation may reflect heightened immune activation or vascular stress in the decidua. In addition, the expression level of the PZP, a known immunomodulator that neutralizes immune activation (148) was downregulated, similar to anti-microbial peptides, important components of the innate immune system at the maternal-fetal interface (149). Similarly, the intensity of the fetal growth regulator PAPPA (150, 151), was also reduced, suggesting an impairment in placental development function. Furthermore, increased levels of hemoglobin subunits (HBA, HBB, HBD) in late RPL compared may result from hyperviscosity/hemoconcentration, which subsequently increases the risk of miscarriage (152, 153). Interestingly, in these studies, low concentration of hemoglobin in the preconception or early pregnancy period, resulting in anemia, also increased the risk of miscarriage (152, 153).

GO analysis in late RPL revealed enriched terms including ‘immune response’, ‘neutrophil degranulation’, ‘platelet activation’, and ‘inflammatory response’, further emphasizing an excessive and dysregulated immune activation, which may contribute to trophoblast stress and placental dysfunction. The presence of oxidative stress-related terms such as ‘cellular oxidant detoxification’, ‘response to reactive oxygen species’, and ‘positive regulation of reactive oxygen species metabolic process’ further supports the notion that placental oxidative stress (123) is a major contributing factor to late RPL. These findings collectively suggest that late RPL is characterized by a pathological environment marked by impaired angiogenesis, dysregulated immune responses, substantial oxidative stress of the trophoblast and placental dysfunction.

### Shared and distinct characteristics in maternal proteome changes in early and late RPL

Among the shared characteristics, we found consistently low levels of CGA across both early and late RPL, indicating a persistent defect in placental function. The pregnancy-specific glycoproteins, such as PSG1, PSG2, PSG4, PSG9, and PSG11, which are essential for immune tolerance by inducing anti-inflammatory cytokines like IL-10 and promoting trophoblast survival (134, 154, 155), were also significantly reduced in both RPL groups. Additionally, we also observed reduced levels of sex hormone-binding globulin (SHBG) in both RPL groups, a key regulator of sex steroid bioavailability. Low SHBG level has been previously associated with early pregnancy loss (67, 156) and not only reflects hormonal or metabolic dysregulation affecting endometrial receptivity and placental development, but may also alter SHBG-mediated estradiol signaling in lymphocytes, potentially disrupting maternal immune adaptation during early pregnancy (157).

However, other findings indicate that immune dysfunction in RPL follows a gestational age-dependent pattern. Early RPL appears to be associated with maternal immune maladaptation characterized by defective complement regulation and insufficient maternal immune tolerance, whereas late RPL shows stronger associations with dysregulation of placental immune function, vascular remodeling, and inflammation. Despite these differences, the persistently low levels of CGA and PSGs across both gestational age groups suggest that placental dysfunction remains a central feature of RPL throughout pregnancy. In addition, in late RPL we found signs of reduced trophoblast invasion and inadequate spiral artery remodeling, which were implicated already in miscarriages (158). The consequently altered placental circulation characterized by turbulent jets damage the villous structure that can also be seen with ultrasound (159). These rheological and mechanical changes also have indirect molecular effects (160), as evidenced by oxidative stress triggered pathological processes (90). Our findings are also consistent with oxidative stress-related molecular changes in the placenta, as reflected in the maternal circulation.

In summary, our results highlight distinct shifts in the maternal immune proteome before and after the establishment of placental circulation. The distinct and overlapping enrichment patterns across gestational ages underscore the dynamic interplay between immune adaptation, placental function, and vascular remodeling during pregnancy, offering insight into the molecular pathways underlying early and late RPL. While these proteomics results offer valuable biological insights and identify potential diagnostic biomarkers, also validated by immunoassay in a small sample, they are exploratory in nature and require further independent validation in larger, external cohorts to confirm their robustness and clinical relevance.

### Strengths and limitations of the study

This study has several strengths, including the use of next-generation proteomics with stringent clinical definitions and homogenous patient groups, enabling the identification of gestational age-specific molecular pathways in RPL. Robust methodologies, such as depletion of abundant proteins, mass spectrometry with stringent FDR controls, and validation through immunoassay correlation, enhance the reliability of findings. Indeed, the correlation coefficient between the mass spectrometry and immunoassay results for PAPPA was outstandingly high, while for CGB it was slightly lower, however, even this is entirely sufficient and consistent with the literature (161).

The limitations of the study include the timing of sample collection at the point of miscarriage, which prevents assessing the predictive value of biomarkers before clinical symptoms. The small sample size which inherently reduces statistical power and increases the risk of both type I (false positive) and type II (false negative) errors, and the use of elective termination pregnancies as controls may limit the generalizability of results, while other potential confounders, such as environmental or lifestyle factors, were not addressed. While our use of multiple testing correction, and cross-validation methods aimed to mitigate these risks, the possibility of overfitting and spurious associations cannot be fully excluded. Additionally, the RPL population in our study primarily consisted of women with two or three prior losses, which represents the lower end of the clinical spectrum. It remains to be investigated whether proteomic alterations are more pronounced or distinct in cases with higher-order losses, which may reflect more severe or recurrent pathological processes.

Since one potential confounder that could influence protein levels is maternal age, which was higher in the RPL group, we investigated the potential effect of this confounder by looking into the data focusing on age-related changes in the proteome. It has been described that aging-related changes in the plasma proteome generally occur over much longer timeframes (i.e., decades) and typically result in modest fold changes (162). For instance, in the case of GDF15 - showing the strongest known association between protein abundance and aging from 1301 proteins - the log_2_ fold change per year of age (β = 0.018) corresponds to only ~1.065-fold change over five years (163). This is minimal compared to the ≥1.5-fold changes observed for DA proteins in our study. Furthermore, correlation analysis between maternal age and proteomic data revealed no significant associations after correcting for multiple comparisons. These findings suggest that maternal age is unlikely to be the primary driver of the observed group differences.

## Conclusions

This study provides critical insights into the molecular pathways underlying RPL by leveraging next-generation proteomics and a gestational age-specific approach. We identified distinct and overlapping DA proteins in early and late RPL, highlighting key biological processes such as ‘placental function’, ‘immune modulation’, ‘oxidative stress’, and ‘vascular remodeling’. Several novel biomarkers showed outstanding discriminative performance and may facilitate a better understanding of RPL pathogenesis. The findings emphasize the importance of gestational age in RPL pathogenesis, with early losses mainly linked to immune dysregulation and placental establishment and late losses predominantly associated with oxidative stress and circulatory changes. The study also underscores the significance of placenta-specific proteins as potential liquid biopsy biomarkers for RPL. While the results demonstrate the promise of proteomics in advancing the understanding and diagnosis of RPL, future research with larger cohorts and longitudinal designs will be essential to validate these biomarkers and to test drugs for the modulation of the identified disease pathways and the prevention of RPL. Our findings highlight the importance of the maternal immune system in maintaining successful pregnancy and suggest that targeting immune pathways may offer novel therapeutic approaches for RPL.

## Data Availability

The raw data supporting the conclusions of this article will be made available by the authors, without undue reservation.
